# Successful Eradication of Hepatitis-C Virus with Sofosbuvir based Antiviral treatment results in improvement in quality of life in cirrhotic patients

**DOI:** 10.12669/pjms.38.4.4537

**Published:** 2022

**Authors:** Bushra Ali, Ikram Ul Haq Raja, Asad Choudhry, Arif Amir Nawaz

**Affiliations:** 1 Dr. Bushra Ali, FCPS, Department of Gastroenterology, Fatima Memorial Hospital, Lahore, Pakistan; 2 Dr. Raja Ikram Ul Haq, FCPS, Department of Gastroenterology, Fatima Memorial Hospital, Lahore, Pakistan; 3 Dr. Asad Choudhry, FRCP. Parsa Trust. Al-Raee Hospital, Gujranwala, Pakistan; 4 Prof. Arif Amir Nawaz FACG, Department of Gastroenterology, Fatima Memorial Hospital, Lahore, Pakistan

**Keywords:** Hepatitis-C, Cirrhosis, Quality of life, Anti-viral treatment

## Abstract

**Objectives:**

To document improvement in quality of life in patients with Hepatitis-C related cirrhosis after successful eradication of the virus.

**Methods:**

In this observational cohort study conducted at Fatima Memorial Hospital from September 2015 to July 2017, patients with HCV were assessed for improvement in quality of life by using FACIT-F questionnaire. We compared the Quality of life (QOL) score before the start of treatment with DAAs and after achieving SVR12 in various aspects of quality of life including physical, emotional, functional and social well-being.

**Results:**

A total of 71 patients, 52 (73%) were CTP class A, 18 (25%) in B and one (1.4%) in C. The mean score of QOL before AVT was 23.93±7.04 and after achieving SVR it was 36.83±6.36 (P-value <0.001). In the subcategories, score of functional wellbeing, physical well-being and social wellbeing were significantly improved except emotional wellbeing scores. All scores improved across the spectrum of patients in the CTP class A and B. There was only one patient in the CTP-C class.

**Conclusion:**

Chronic HCV infection complicated by cirrhosis causes a significant decline in quality of life. There was a marked improvement in the functional, social and physical health of the patients after eradication of Hepatitis-C with anti-viral therapy except emotional health of the individuals.

## INTRODUCTION

According to WHO estimates, there were 71 million persons living with HCV infection worldwide in 2015 accounting for 1% of the world’s population.^1^ An estimated 6.8% of the general population is infected with HCV in Pakistan and genotype 3a is most predominant genotype.^2^,^3^ Chronic HCV infection leads to cirrhosis to 5 – 25% of patients over a period of two to three decades.^4^ Poorer quality of life has been observed in patients with advanced cirrhosis especially in terms of physical and functional well-being.^5^ There is a marked reduction in all-cause and liver related mortality, development of hepatocellular carcinoma and need for liver transplantation once hepatitis C is eradicated successfully.^6^,^7^

Most of the literature on outcomes of treatment of HCV is focused on liver function, mortality, or incidence of life-threatening complications. Emotional, physical, functional and social well-being and any improvement thereof has not been addressed adequately in the previous studies. There is some evidence to suggest improvement in quality of life in patients who achieve SVR with direct acting antivirals (DAAs).^8^,^9^ In our study we used the FACIT-F generic core questionnaire to assess the functional, physical, emotional and social wellbeing of cirrhotic patients before and after successful eradication of HCV, which is used in previous studies.^10^

## METHODS

An observational cohort study carried out at Department of Gastroenterology, Fatima Memorial Hospital from September 2015 to July 2017. Approval was taken from the Institutional Review Board (Ref: FMH-08-2018-IRB-492-M, Dated: October 29, 2018). Seventy one patients were included using non probability consecutive sampling technique. Patients aged between 18 to 65 years, with compensated & decompensated cirrhosis due to hepatitis C who are undergo HCV treatment were included in the study. Patient with co infection with HBV or HIV, Hepatocellular carcinoma and those who were admitted for an acute episode of upper G.I bleeding, hepatic encephalopathy, spontaneous bacterial peritonitis or hepatorenal syndrome were excluded. Pre-treatment assessment of quality of life was done using FACIT-F questionnaire. The suitable candidates were treated with standard regimens of direct acting antiviral agents after recording their demographics and parameters for Child-Turcotte-Pugh (CTP) class. Patients achieving SVR 12 were assessed, those patients who achieved SVR12 were asked again to fill the FACIT-F questionnaire for assessment of quality of life. There were a total of 18 questions pertaining to physical, emotional, functional and social well-being. Each question had a maximum score of three and a minimum of one. A score of three was perceived as better health than a score of one. Hence the maximum total score which could be attained was 54 and minimum 18. Data analysis was done on SPSS 20 and paired t-test was applied.

## RESULTS

In this study, we compared the quality of life score before the start of treatment with DAAs and after achieving SVR12. A total of 71 patients were enrolled including 44 (62%) women and 27(38%) men. There were 52 (73.24%) patients in CTP class A, 18 (25.35%) in CTP class B and only one (1.41%) in CTP class C. Amongst CTP class A patients mean age was 51.38±9.06 and the average age of CTP class B patients was 52.06±9.37. The only patient in CTP class C was 54 years old.

Fifty-six patients (79%) achieved SVR12 with sofosbuvir and ribavirin, 13 (18%) with sofosbuvir, daclatasvir and ribavirin and two patients with sofosbuvir and ledipasvir (3%). Out of a total of 71 patients, the mean Quality of life score before DAAs treatment was 23.93±7.04 which increased to 36.83±6.36 after achievement of SVR (P-value <0.001). In the sub category of functional wellbeing, difference in perception of physical well-being, scores of social wellbeing also improved significantly before treatment and after achieving sustained virological response. No difference was however noted in the emotional wellbeing score which remained the same before and after treatment. The scores of these subcategories before and after eradication of hepatitis C virus is shown in Table-I.

**Table-I:** Statistically significant improvement was seen in all wellbeing scores except emotional wellbeing with significant improvement of overall QoL scores (n=71).

	Mean score before DAAs	Mean Score after SVR 12	P value
Functional wellbeing	6.43 +/- 2.12	9.80 +/- 2.78	<0.001
Physical wellbeing	7.35 +/-2.52	12.43 +/- 1.85	<0.001
Social wellbeing	2.281 +/- 0.7	2.56 +/- 0.76	<0.001
Emotional wellbeing	12.02 +/- 2.467	12.02 +/- 2.467	-

Out of these 71 patients, 52 were in CTP class A at the time of treatment. In child class A patients, total QoL score rose from a mean of 21.98 +/- 5.34 to 35.84+/- 4.94. The scores of pre and post well beings in subcategories are shown in Table-II. In the subcategory of functional wellbeing, physical well-being and social wellbeing showed significant improvement in pre and post treatment analysis. Emotional wellbeing, however, remained the same after treatment.

**Table-II:** Amongst patients with Child Class A cirrhosis, statistically significant improvement was seen in all wellbeing scores except emotional wellbeing with significant improvement of overall QoL scores (n=52).

	Mean score before DAAs	Mean Score after SVR 12	P value
Functional wellbeing	5.94+/- 1.68	9.30+/- 2.52	< 0.001
Physical wellbeing	6.69+/- 2.05	12.07+/- 1.51	<0.001
Social wellbeing	2.269+/- 0.59	2.53+/- 0.699	<0.001
Emotional wellbeing	11.46+/- 1.95	11.46 +/- 1.46	-

There were 18 patients in CTP class B. The QoL score for functional wellbeing, physical wellbeing, perception of social wellbeing score significantly improved after treatment, while score for emotional wellbeing remain unchanged. In child class B patients, total QoL score rose from a mean of 27.88±6.05 to 40.056±7.657.

## DISCUSSION

Chronic liver disease like other chronic diseases has significant impact on physical as well as mental health.^11^,^12^ Chronic hepatitis C infection, regardless of the degree of fibrosis, affects the quality of life of infected individuals.^13^,^14^ It is important to know improvement in different parameters of quality of life after successful eradication of the virus. Different tools have been used to assess the quality of life in these patients including FACIT-F, SF-36 questionnaire, CLDQ-HCV (Chronic Liver Disease Questionnaire - Hepatitis C Version), WPAI:SHP (Work Productivity and Activity Impairment - Specific Health Problem).^15^ Using FACIT-F, in our study, there was significant improvement in health-related quality of life in patient who achieved SVR (p value <0.001). Our results are consistent with the studies addressing this issue.^16^,^17^ Our study endorsed the results presented by Morad et al and Xie Q et al in Egyptian and Chinese populations respectively.^18^,^19^ Similarly, Kesen O et al have the similar results in Turkish populations.^20^ Contrary to our results, Thuluvath PJ et al showed only marginal improved in QoL after eradication of HCV. The results may be different because of different population with different socioeconomic status.^21^ A study by Younossi Z et al, however, showed worsening of QoL parameters in patients who were unable to achieve sustained virological response after a course of DAAs, which further strengthens our results.^22^

Total baseline FACIT scores were low in our study as compared to the international study probably because of difference in socioeconomic status of the populations observed in these studies.^15^ In our study, there was improvement in all other parameters except the emotional wellbeing. Previous studies however did show improvement in this category also.^23^,^24^ The reasons for this difference could be multifactorial including lack of resources for ongoing medical care, lack of family support due to concerns about infectivity as well as economic challenges. This is an important indicator of overall well-being and should be addressed in future studies.

Improvement was seen in both CTP class A & B, which is consistent with the study done by Ioannou G et al have previously shown significant improvement in QoL scores across the spectrum of CTP class patients.^25^ Our study also shows improvement in QoL score in CTP class A & B patients. It is difficult to comment on CTP class C patients as only one patient of this category was included in our study, however Zobair M et al found that there was more improvement in decompensated cirrhosis patients as compared to compensated ones.^26^

**Table-III:** Amongst patients with Child Class B cirrhosis, statistically significant improvement was seen in all wellbeing scores except emotional wellbeing with significant improvement of overall QoL scores (n=18).

	Mean score before DAAs	Mean Score after SVR 12	P value
Functional wellbeing	7.389 +/- 2.033	10.94 +/- 2.999	<0.001
Physical wellbeing	8.83 +/- 2.54	13.33 +/- 2.38	<0.001
Social wellbeing	2.111 +/- 0.323	2.444 +/- 0.51	<0.001
Emotional wellbeing	13.333 +/- 2.95	13.333 +/- 2.95	-

### Limitations:

It includes the retrospective nature of the study and smaller sample size. Other comorbidities affecting quality of life have not been taken into account therefore larger prospective studies are needed to address this very important issue.

## CONCLUSION

Chronic HCV infection complicated by cirrhosis causes a significant decline in quality of life by its impact on physical, emotional, social and functional wellbeing of the patient. Our study shows significant improvement in overall QoL scores after treatment of HCV infection leading to SVR 12. Larger prospective studies are needed to address this important issue.

### Authors’ Contribution:

**BA** conceived, designed and did data analysis & editing of the manuscript. She is also responsible for the accuracy or integrity of the study.

**AC and RIH** did data collection and manuscript writing. **AAN** did review and final approval of the manuscript.
